# Primary Care Referrals of Suspected Eating Disorders in Children and Young People in Greater Manchester Audit

**DOI:** 10.1192/bjo.2022.84

**Published:** 2022-06-20

**Authors:** Benjamin Geers, David Ochando

**Affiliations:** 1 University of Manchester, Manchester, United Kingdom; 2 Manchester University NHS Foundation Trust Community Eating Disorders Service, Manchester, United Kingdom

## Abstract

**Aims:**

Current NICE guidance states that Children and Young People (CYP) with suspected Eating Disorders (EDs) should be immediately referred to specialist services on their first presentation to primary care. This audit assessed whether these standards were being met across Greater Manchester and what general practitioners felt would be helpful in supporting them to correctly refer patients. *Aim 1:* Analyse information from referral forms on all patients referred to Manchester Foundation Trust Community Eating Disorders Service (MFT-CEDS) from primary care between 17th December 2020 – 17th June 2021. *Aim 2:* Gain insight into how confident GPs in Greater Manchester feel identifying suspected EDs in CYP, their knowledge of guidelines regarding referral, and how they would like to be supported to improve referrals of suspected CYP-ED.

**Methods:**

Quantitative data on all primary care referrals made between 17th December 2020–17th June 2021 were analysed. Referrals were classified as correct if they were made both immediately and directly to the correct service. Subgroup analysis of data by geographic region of Greater Manchester was also undertaken. Qualitative data were collected through a survey which was sent to General Practitioners across Greater Manchester. The survey assessed knowledge of current guidelines and views on what training materials could be helpful to improve the referral process.

**Results:**

A total of 69 patients were referred to MFT-CEDS by their GP between 17th December 2020 and 17th June 2021. 35% of GP referrals to MFT-CEDS were documented as being made correctly as per current guidelines. 43.5% of all referrals were not initially made to MFT-CEDS. 58% of referrals were documented as being made immediately. North and South Manchester had the lowest rates of correct referrals of 10% and 8% respectively. There were 10 survey respondents, of which the majority did not know current referral guidelines and did not feel confident in identifying suspected Eating Disorders in CYP.

**Conclusion:**

The majority of primary care referrals of CYP with suspected eating disorders to MFT-CEDS were not made in line with current NICE guidance.

The following recommendations were made based on the findings of this audit:

1) Create an information document and video regarding identification and referral guidelines for suspected EDs in CYP, 2) Design an easy-to-use referral template for GPs, 3) Conduct interviews with GPs working in North and South Manchester to help identify what additional support they need, 4) Re-audit referral data once quality improvement measures have been in place for 6 months.

